# Rapid methods for the evaluation of fluorescent reporters in tissue clearing and the segmentation of large vascular structures

**DOI:** 10.1016/j.isci.2021.102650

**Published:** 2021-05-26

**Authors:** Nils Kirschnick, Dominik Drees, Esther Redder, Raghu Erapaneedi, Abel Pereira da Graca, Michael Schäfers, Xiaoyi Jiang, Friedemann Kiefer

**Affiliations:** 1 European Institute of Molecular Imaging, University of Münster, Waldeyerstraße 15, 48149 Münster, Germany; 2 Institute of Computer Science, University of Münster, Einsteinstraße 62, 48149 Münster, Germany

**Keywords:** Optical imaging, computational bioinformatics

## Abstract

Light sheet fluorescence microscopy (LSFM) of large tissue samples does not require mechanical sectioning and allows efficient visualization of spatially complex or rare structures. Therefore, LSFM has become invaluable in developmental and biomedical research. Because sample size may limit whole-mount staining, LSFM benefits from transgenic reporter organisms expressing fluorescent proteins (FPs) and, however, requires optical clearing and computational data visualization and analysis. The former often interferes with FPs, while the latter requires massive computing resources. Here, we describe 3D-polymerized cell dispersions, a rapid and straightforward method, based on recombinant FP expression in freely selectable tester cells, to evaluate and compare fluorescence retention in different tissue-clearing protocols. For the analysis of large LSFM data, which usually requires huge computing resources, we introduce a refined, interactive, hierarchical random walker approach that is capable of efficient segmentation of the vasculature in data sets even on a consumer grade PC.

## Highlights

• 3D-polymerized cell dispersions allow evaluation of tissue clearing protocols
• Protein fluorescence retention can be tested during tissue clearing
• A semi-automated random walker enables segmentation of memory exceeding image data

## Graphical abstract

**Figure:**
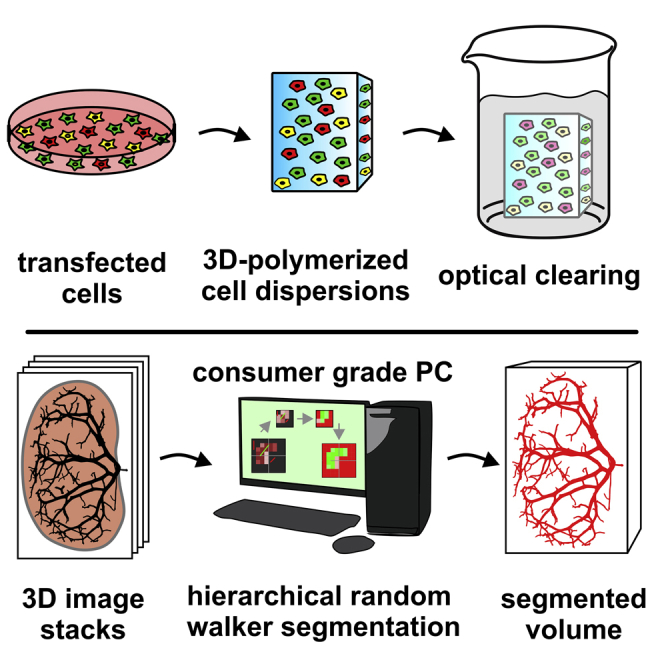

## Introduction

Confocal laser scanning (CLSM) and light sheet fluorescence microscopy (LSFM), two optical sectioning microcopy techniques, have become mainstay in biological and biomedical research. Over the last two decades, spatial resolution and sensitivity of confocal microscopes improved significantly (Bayguinov et al., 2018; Jonkman et al., 2020) requiring however high numerical aperture objectives, inextricably associated with limited working distance. This impelled the development of alternative technologies for imaging of large tissue volumes, most notably LSFM. Initially conceived a century ago, LSFM experienced since its reintroduction in 2004 a massive renaissance (Huisken et al., 2004; Siedentopf and Zsigmondy, 1902). In LSFM, the sample is illuminated with a thin sheet of light, while signals from this illuminated plane are recorded by an orthogonally oriented second objective (Power and Huisken, 2017). By moving the light sheet stepwise through the sample, stacks of images, each representing one sample plane, are recorded. Use of long-working distance detection optics makes LSFM ideal for the analysis of spatially complex structures in large tissue volumes, acquisition of which typically requires stitching of multiple tiled stacks with each stack often being composed of several thousand planes. Resulting data sets may range from several hundred GB to TBs in size and thus require the development of new approaches for stitching, rendering, and quantitative evaluation. First elegant solutions have been developed but require high powered computing (Pietzsch et al., 2015; Bria et al., 2019; Bria and Iannello, 2012; Kirst et al., 2020; Hägerling et al., 2017; Schoppe et al., 2020; Todorov et al., 2020). Therefore, there is an unmet need for approaches that allow the quantitative analysis of large multiscale data sets using widely available commodity computing hardware. Currently, many approaches do not consider and thus do not support the analysis of out-of-core (larger than main memory) data sets, particularly for complex image analysis where problem formulations are often global and cannot be solved by local computations, which only consider a small subset of the image. This is especially problematic, as the historic trend of exponential decrease in price per memory unit appears to change in recent years (McCallum et al., 2020).

Except for small translucent samples, LSFM requires optical clearing to reduce scattering, which prevents the light sheet to penetrate opaque tissue due to lipid constituents and small vesicles within cells (Richardson and Lichtman, 2015). Already a century ago, Spalteholtz developed the first protocol to render anatomical specimen translucent (Spalteholz, 1914), providing an essential road map for tissue clearing. Following extraction of lipids and water (delipidation/dehydration), the sample is incubated in a fluid of roughly the refractory index (RI) of protein, referred to as RI matching. In biomedical research, LSFM is nearly exclusively focused on the detection of fluorescent signals that originate either from staining with dye-labeled high affinity binders, mostly antibodies or nanobodies, or by expression of one or more of the ever increasing palette of fluorescent proteins (Shaner et al., 2005). Alternatively, protein tags can bind fluorophores or convert non-fluorescent dyes to a fluorescent form (Rodriguez et al., 2017; Peresse and Gautier, 2019; Bolbat and Schultz, 2017). While LSFM allows the non-destructive analysis of large samples, whole-mount staining crucially depends on full sample penetration by the staining reagent. Genetically encoded, fluorescent proteins are expressed throughout the entire sample, but while fluorescent dyes or fluorogens are mostly insensitive to dehydration, proteins rapidly go dark in organic solvents (Orlich and Kiefer, 2018). To address this issue, a number of tissue clearing technologies, aiming to retain reporter fluorescence, were developed. Based on the chemical environment during delipidation and RI matching, organic-solvent-based and hydrophilic protocols are distinguished (Ueda et al., 2020; Matryba et al., 2019). Due to their high capacity to dissolve lipids, organic-solvent-based protocols provide excellent tissue clearing and the associated dehydration often results in shrinkage and stabilization of the sample. On the other hand, fluorescent proteins evolved in an aqueous environment and are sensitive to the denaturing action of organic solvents. Several approaches were developed to overcome this obstacle, including the use of higher-order organic alcohols for delipidation and RI matching at alkaline pH (Schwarz et al., 2015; Klingberg et al., 2017; Qi et al., 2019). More recently, addition of polyethylengylcol during delipidation and RI matching was described (Jing et al., 2018). While variable results have been reported for these approaches, hydrophilic clearing protocols generally retain fluorescence better but tend to produce expanded, less stable samples, often displaying inferior transparency (Orlich and Kiefer, 2018). The multitude of tissue clearing protocols and an increasing number of newly described fluorescent proteins, which are often insufficiently characterized, make the selection of the most optimal combination for a particular question difficult and highlight the need for reliable prior testing.

Here, we introduce 3D-polymerized cell dispersions, a fast and straightforward tool, which is based on recombinant expression of a protein of choice in a freely selectable tester cell line, to evaluate and compare tissue-clearing protocols. The underlying aim was to develop a simple methodology, resembling a generic tissue in the best possible way. This tissue surrogate should be generated rapidly, be of low cost, and involve materials routinely used in cell biology laboratories. Results obtained by this approach were verified by analysis of a reporter mouse line for lymphatic vessels. Retention of protein fluorescence after tissue clearing of different organs derived from adult mice or midgestation fetuses is demonstrated. Finally, we introduce a refined, interactive, hierarchical random walker approach to demonstrate efficient segmentation of vascular structures with radii of multiple orders of magnitude on a consumer grade PC. The vessel analysis, which was performed on large data sets by far exceeding the available memory, has a single, dimensionless pruning parameter that enables processing of multiscale data sets.

## Results

### Preparation of 3D-polymerized cell dispersions

**Figure 1:**
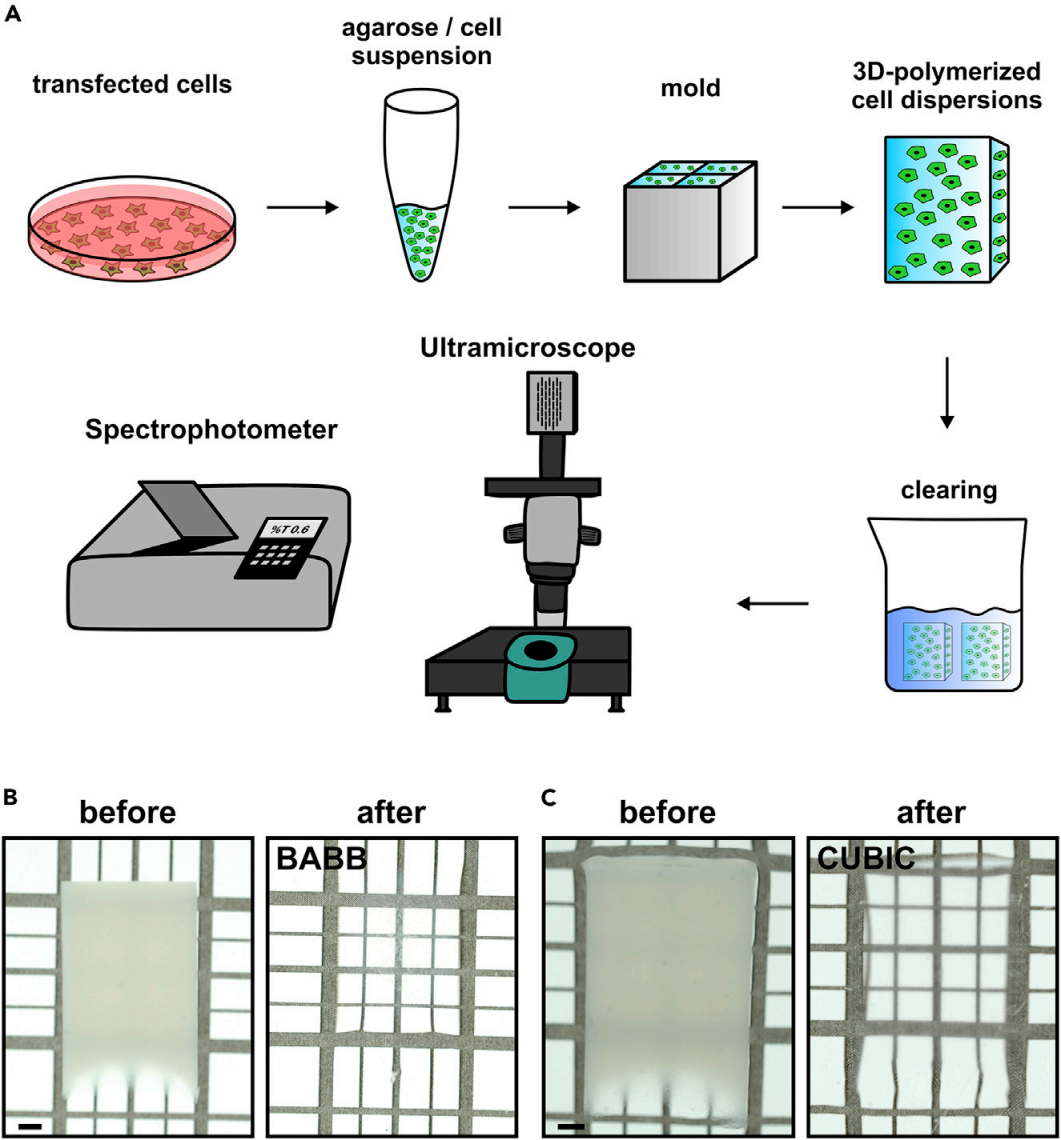
Workflow for rapid testing of the compatibility of tissue clearing protocols and fluorescent proteins or fluorogen-based reporters (A) Transfected cells stably or transiently expressing one or more selected fluorescent proteins or protein tags are formaldehyde fixed, harvested, and resuspended in low melting agarose. After solidification in a mold, the agarose block with the cells is extruded, wholemount stained where required, and subjected to tissue clearing following various protocols. At various time points before, during or after completion of refractory index matching a surface proximal volume of 500 μm depth of the agarose block is imaged using light sheet microscopy to determine fluorescence retention. At the same time, transmission is determined in a spectrophotometer. (B and C) Transillumination picture of an agarose block placed on a test grid before and after clearing in methanol/BABB (B) or following the CUBIC protocol (C). Scale bar represents 1mm.

Basis for the 3D-polymerized cell dispersions, which we describe here, is the expression of one or more fluorescent proteins of choice in a eukaryotic cell line. Any cell line suitable for transfection may be used. We selected HEK 293T cells for our exemplary experiments, as they are simple to grow, easy to transfect, and highly proliferative, allowing the rapid expansion to large cell numbers (Figure 1A). Stably transfected HEK 293T cells were briefly tested for expression of the desired fluorescent proteins, detached from the cell culture dish, washed, and fixed. We selected low melting agarose as matrix for the generation of 3D-polymerized cell dispersions, as it is readily available in many labs, easy to handle, and compatible with cellular integrity and a wide range of clearing protocols. A formaldehyde-fixed cell suspension was adjusted to approximately 10 x 10^6^ cells/ml and then mixed with 2% low melting agarose in a 1:1 ratio. Depending on the desired properties, e.g., FP expression level, cell density in the mixture may be adjusted within a wide concentration range, we used between 1,000 and 10,000 cells/mm3. The cell-agarose mixture was then allowed to cure in a custom mold (see STAR Methods section), and these newly formed cell-agarose blocks were extruded and stored in phosphate-buffered saline until further usage.

An obvious dilemma for the assessment of fluorescence retention during tissue clearing is posed by the initially limited transparency. However, the described agarose-based tissue surrogates offered sufficient light penetration to allow quantitative fluorescence measurement in a shallow, surface-proximal cubic volume of 500 μm depth, which was analyzed by light sheet microscopy at various times during the clearing process. At the same time, optical transparence was measured with a spectrophotometer (Figure 1A). Transillumination photographs of two representative 3D-polymerized cell dispersions before and after organic (benzoic acid/benzyl benzoate [BABB], Figure 1B) or hydrophilic (clear, unobstructed brain/body imaging cocktails [CUBIC] Figure 1C) clearing demonstrate the suitability of the model.

### Comparison of fluorescence preservation and clearing performance of four different clearing protocols using 3D-polymerized cell dispersions

We tested 3D-polymerized cell dispersions of HEK 293T cells transiently transfected with expression plasmids for the enhanced green fluorescent protein (EGFP) or mOrange2 or mCherry or tdTomato, all of which are widely used as reporter proteins for monitoring gene expression, protein localization, and as molecular sensors (Shaner et al., 2004, 2005). 3D-polymerized cell dispersions were cleared following the BABB (Dent et al., 1989; Becker et al., 2008), ethyl cinnamate (ECi) (Klingberg et al., 2017), polyethylene glycol (PEG)-associated solvent system (PEGASOS) (Jing et al., 2018), and CUBIC (Susaki et al., 2014) protocols. All three organic-solvent-based protocols, BABB, ECi, and PEGASOS employ incubation in ascending alcohol concentrations for delipidation and dehydration before RI matching and, however, employ different alcohols. In contrast, CUBIC-based tissue clearing achieves decolorization by incubation in amino alcohols, non-ionic detergent, and urea-mediated hyperhydration, homogenizing the cellular microenvironment and decreasing scatter.

**Figure 2:**
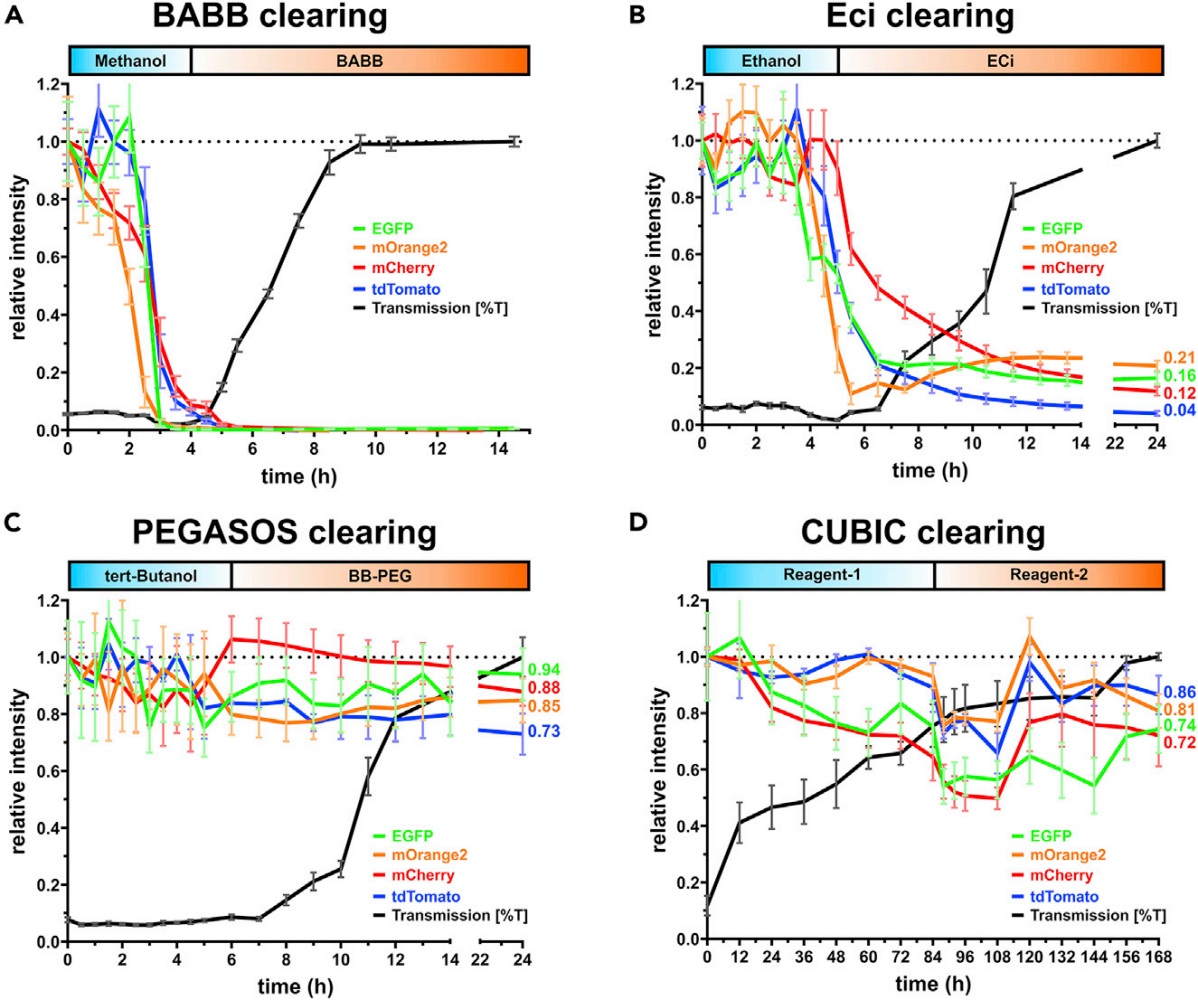
Sample transparency and fluorescence retention during different tissue clearing procedures HEK293T cells stably expressing the indicated fluorescent proteins were embedded in agarose and subjected to different tissue clearing protocols as outlined in Figure 1A, the respective dehydration/delipidation and RI matching solutions as well as the duration of their application are indicated by the blue and orange boxes on top. (A) Dehydration/delipidation with methanol followed by refractory index matching in benzoic acid/benzyl benzoate (BABB). (B) Dehydration in buffered ethanol (pH 9.0) followed by refractory index matching in ethyl-3-phenylprop-2-enoate (ethyl cinnamate, ECi). (C) Dehydration/delipidation in tert-butanol/Quadrol followed by refractory index matching in benzyl benzoate/PEG-MMA-500 (BB-PEG) according to the PEGASOS protocol. (D) Delipidation in Quadrol, urea, and Triton X-100 (reagent 1) followed by refractory index matching in sucrose, urea, and triethanolamine (reagent 2) according to the CUBIC protocol. Fluorescence intensities for the different proteins at t = 0 were normalized to 1, and colored line plots denote the relative fluorescence intensity of the indicated proteins in four independent samples (mean ± SEM). The black line denotes the transmission (mean ± SEM) at the indicated time points in four individual samples.

As is immediately obvious from Figures 2A and 2B and has been reported previously, the denaturing effect of the dehydration in methanol and ethanol causes a loss of fluorescence of all four proteins. This effect is rapid and complete in methanol and happens slower and less uniform in alkaline ethanol used in the ECi protocol. In both cases, mOrange2 was most sensitive to dehydration while mCherry showed the highest resilience to ethanol. Its loss of brightness in ECi, however, continued until a stable residual fluorescence of 12% was reached. As described by Giese and coworkers (Schwarz et al., 2015), tert-butanol provided improved fluorescence retention (Figure 2C). Furthermore, addition of modified PEG and Quadrol in the PEGASOS protocol (Figure 2C) further contributed to structural stabilization, to radical quenching, and to the maintenance of an alkaline pH. Overall, 70% or higher fluorescence retention after PEGASOS clearing was observed. Optical transparency was most efficiently obtained in all organic RI matching solutions. An increase in transparency was rapidly noticeable after incubation in BABB, reaching completeness after 5 hr of RI matching (Figure 2A). In ECi and BB-PEG, sample transparency was first detectable after approximately 2 hr and was complete less than 20 hr later (Figures 2B and 2C). In contrast, samples took 7 days for clearing in CUBIC, which however retained fluorescence at 70% or higher (Figure 2D).

Taken together, 3D-polymerized cell dispersions provided an excellent tool to investigate the progress of clearing and associated fluorescence loss during different protocols with unprecedented temporal resolution. The high degree of accordance with published studies on tissue clearing confirmed the validity of the data obtained by analysis of 3D-polymerized cell dispersions and underscored the suitability of this universal tester system.

### Comparison of four different clearing protocols on diaphragm tissue samples

Next, we aimed to verify our previous results using biological samples. Because the fluorescence of tdTomato had been persistently the most stable of the proteins tested, we decided to analyze an *mVegfr3*-tdTomato reporter mouse model. In these animals, tdTomato is expressed as a bacterial artificial chromosome (BAC) transgene under the control of murine *Vegfr3* promotor elements, giving rise to more moderate expression levels compared to transfected cell lines.

**Figure 3:**
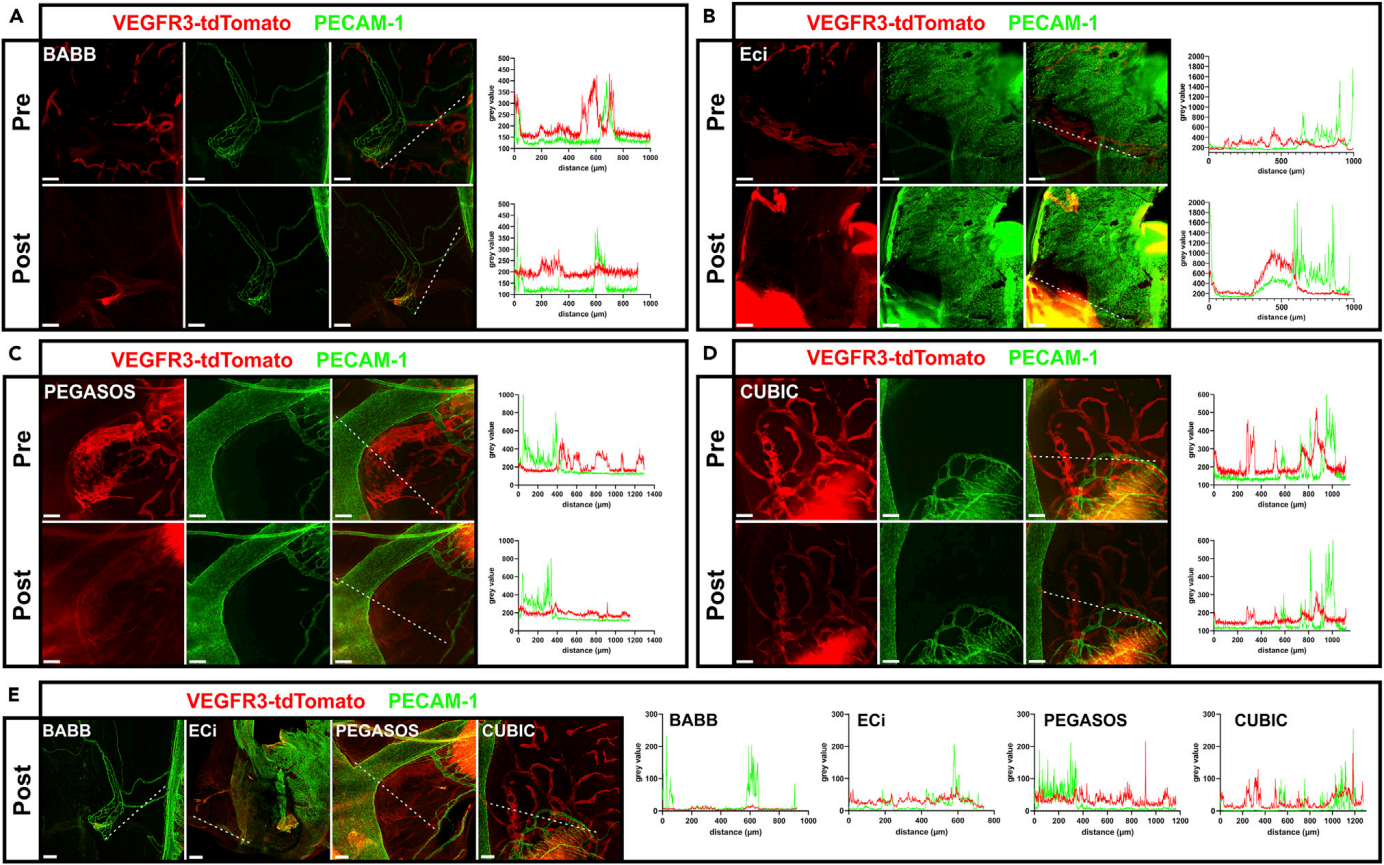
Fluorescence retention after clearing of the diaphragm of an m*Vegfr3*-tdTomato reporter mouse (A–D) Four samples from the fixed diaphragm of an m*Vegfr3*-tdTomato reporter mouse were cleared following the tissue clearing protocols described in Figure 2. Top and bottom rows show comparable micrographs of view fields before (pre) and after (post) clearing acquired by epifluorescence microscopy using LED illumination. Contrasting of the vascular system by staining with Alexa Fluor 647-labeled anti-PECAM1 antibodies served to provide unambiguous anatomical landmarks for orientation. (E) After epifluorescence imaging, the cleared, mounted samples were reanalyzed using CLSM, and merged panels corresponding to the view fields in (A–D) are shown as maximum intensity projections (MIPs). Intensity profiles (gray value) of tdTomato (red) and PECAM-1 (green) expression along the white dashed line are presented in the graph on the right. Scale bars represent 150μm.

Like all vessels, the tdTomato expressing lymphatic vessels in this mouse model are formed by a single layer of thin endothelial cells; hence, their visualization requires both optimal fluorescence retention and efficient tissue clearing. To provide stable anatomical landmarks and to judge the quality of clearing, the blood vasculature in the sample was marked by Alexa Fluor 647-coupled anti-PECAM1 antibodies. Samples were analyzed by epifluorescence and CLSM or LSFM. A well-suited organ for the analysis is the diaphragm, in which blood and lymphatic vessels run in planar orientation between the underlying muscle and mesothelial cells, which only form a thin epithelial layer. The diaphragm of an anti-PECAM1-Alexa Fluor 647-labeled 10-week-old *mVegfr3*-tdTomato mouse was explanted, formaldehyde fixated, and divided into four parts, which were briefly imaged using an epifluorescence microscope with light-emitting diode (LED) illumination (Figures 3A–3D top panels, labeled before). Subsequently, samples were subjected to BABB, ECi, PEGASOS, and CUBIC clearing, precisely following the protocols as applied for the analysis of HEK cells.

After completion of clearing, samples were again imaged using the epifluorescence microscope (Figures 3A–3D bottom panels, labeled after). Comparable view fields were identified using blood vessels as positional landmarks, changes in the relative position of these landmarks were caused by sample shrinkage or swelling following dehydration or hyperhydration (Figures 3A–3D; PECAM-1) (Orlich and Kiefer, 2018). Binding and fluorescence of Alexa Fluor 647-labeled anti-PECAM-1 antibodies, here used to delineate the blood vessels, are not affected by tissue clearing (Dierkes et al., 2018; Pollmann et al., 2014). The massive increase of background fluorescence detected in the red channel was caused by the diaphragm muscle layer, which only became optically accessible after tissue clearing, while it was hidden by scattering in uncleared tissue. In good agreement with the data obtained from clearing of 3D-polymerized cell dispersions, tdTomato fluorescence was quenched after BABB and ECi clearing (Figures 3A and 3B, bottom panels labeled after). The marked increase in red autofluorescence after ECi clearing resulted from pronounced sample deformation, which prevented completely flat mounting of the specimen (Figure 3B, bottom panels). Consequently, the muscle and vasculature were acquired in the same optical plane. In keeping with the results obtained after clearing of 3D-polymerized cell dispersions, fluorescence retention was superior in the samples cleared by the PEGASOS and CUBIC protocols, where now sufficient signal intensity was retained to keep the lymphatic vasculature discernible. Still, signal intensity was weaker following the PEGASOS protocol compared to CUBIC-cleared samples, which slightly differ from our assessment using 3D cell dispersions (Figure 3C bottom panels). To investigate if this discrepancy was primarily caused by the low tdTomato expression level, which is characteristic for the *mVegfr3*-tdTomato reporter mouse, we tested fluorescence retention after PEGASOS clearing of 3D cell dispersions containing HEK293T cells that either expressed low or high levels of EGFP or tdTomato (Figure S1). Interestingly, the relative fluorescence intensity normalized to fluorescence before clearing retained from EGFP and tdTomato high expressing cells was slightly lower compared to the fluorescence retained from cells expressing lower levels of these fluorescent proteins (Figure S1C). This was most likely due to a more efficient detection of weakly fluorescing cells as clearing progressed, suggesting that the observed differences were not due to tdTomato expression levels. We therefore decided to test fluorescence retention of a different protein and additionally probed diaphragm samples from Lifeact-EGFP reporter mice (Figure S2). While also EGFP fluorescence in tissue samples of Lifeact-EGFP mice was reduced after PEGASOS clearing, reduction appeared to occur to a lesser extent compared to *mVegfr3*-tdTomato fluorescence (Figures S2A and 3C). By far, the best signal retention of tdTomato was observed after CUBIC clearing, where the entire *Vegfr3-tdTomato*-positive lymphatic vessel bed was fully delineated (Figure 3D, bottom panel labeled after). Due to higher illumination light intensity and far thinner optical sections, CLSM provides a more efficient background exclusion and weak signal detection. Therefore, we reanalyzed the samples by CLSM (Figure 3E and S2B), which confirmed the findings by epifluorescence microscopy.

In summary, the rapid judgment obtained using 3D-polymerized cell dispersions regarding compatibility of tissue clearing and fluorescent proteins allowed a good prediction of these factors during the analysis of a transgenic model system, thereby validating our approach.

### Clearing and light sheet imaging of large tissue volumes

**Figure 4:**
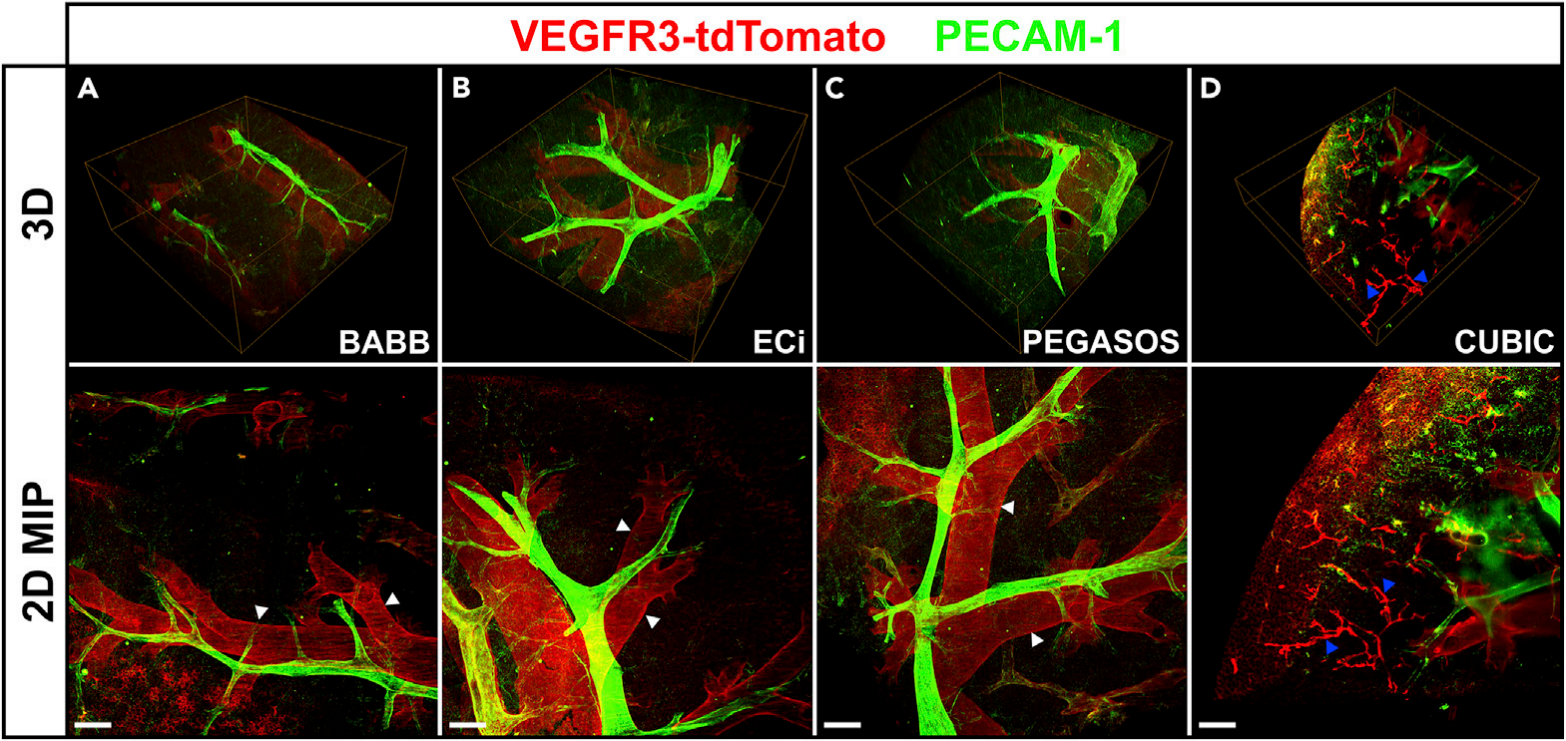
Fluorescence retention after clearing the lung of an m*Vegfr3*-tdTomato reporter mouse The lung of an m*Vegfr3*-tdTomato reporter mouse was explanted, fixed and four tissue blocks were derived from the organ and subjected to the clearing procedures as outlined in Figure 2 . Again the blood vasculature was contrasted with Alexa Fluor 647-labeled anti-PECAM1 antibodies. Image stacks of the cleared tissues were acquired by light sheet microscopy. Panels in the top row depict MIPs of digital three dimensional volume reconstructions of the complete cleared tissue blocks using the BABB (A), ECi (B), PEGASOS (C) and CUBIC (D) tissue clearing protocols. Dimensions: 3.02 × 3.02 × 1.20 mm (A-C) and 0.83 mm for (D). Panels on the bottom row depict axial (z axis) MIPs of the same volumes in the upper panels. Only CUBIC clearing preserved the fluorescence of tdTomato to a notable degree (e.g. blue arrowheads in [D]). Note the strong red (602–662 nm) autofluorescence of the bronchi following the treatment with organic solvents (white arrowheads). Scale bars in the bottom row equal 300 μm in the x/y plane.

The diaphragm is a comparatively thin tissue, well suited for microscopic analysis after flat mounting. To test the clearing procedures on a significantly larger tissue volume, we applied them to lung samples of approximately ~2 × 2 × 2 mm that were prepared from the resected left lobe of an *mVegfr3*-tdTomato mouse. Again, Alexa Fluor 647-labeled anti-PECAM-1 antibody staining provided anatomical landmarks and analysis was done by LSFM. Volume renderings of the complete tissue block are depicted in the top panel of Figure 4, while aspects of magnified subvolumes are shown in the bottom panel. Solvent-based clearing resulted in a strong red autofluorescence of the trachea and bronchi, likely originating from elastic and cartilaginous components. Discernible lymphatic vessels were exclusively detectable in CUBIC-cleared samples, confirming our previous notion that CUBIC is superior in maintaining the fluorescence of tdTomato protein to organic solvent-based protocols.

**Figure 5:**
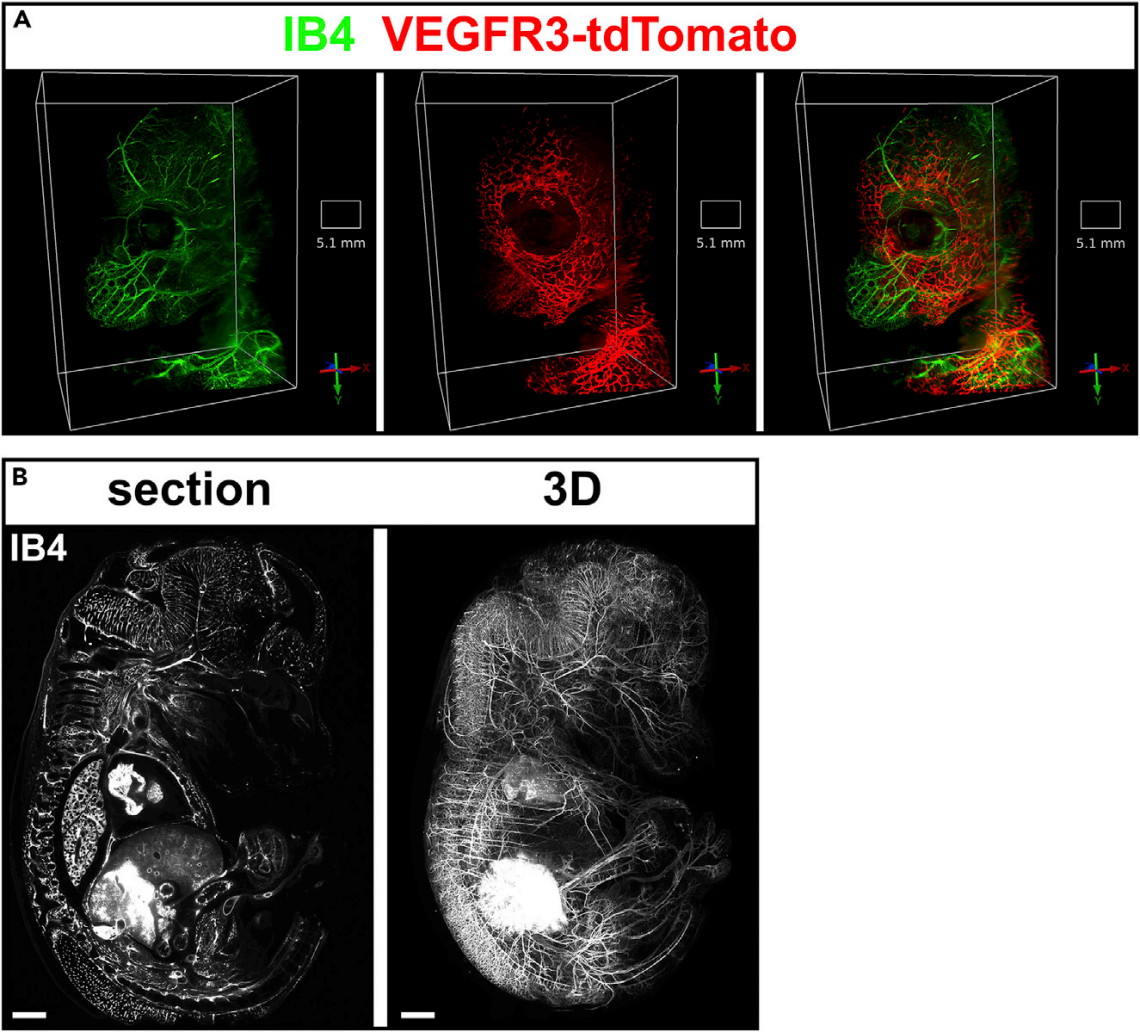
Light sheet microscopic visualization of developing lymphatic vessels in the head of an m*Vegfr3*-tdTomato E14.5 mouse fetus (A) Volume reconstruction of the developing cranial blood (contrasted by IB4-Alexa Fluor 647, green) and lymphatic vessels (endogenous fluorescence, red) in the head of an *mVegfr3*-tdTomato E14.5 mouse fetus after tissue clearing following the CUBIC protocol. Square base scale of each reconstruction with its size is depticed in the each image. (B) Light sheet-based 3D visualization of the IB4-Alexa Fluor 647-contrasted entire blood vasculature of an E14.5 mouse fetus cleared by dehydration/delipidation in methanol and subsequent RI matching in BABB. The left panel depicts a single sectional plane of 3 μm thickness, whereas in the right panel, an MIP of the digital three dimensional volume reconstruction is shown (dimensions: 10.4 × 12.4 × 5.3 mm), scale bar represents 400 μm.

Having demonstrated the capacity of CUBIC to clear larger tissue blocks and to retain sufficient signal from the single layered lymphatic endothelial cells in *mVegfr3*-tdTomato mice to image the lymph vasculature, we aimed to extend this to cm^3^ volumes by analysis of a late midgestation mouse fetus at the developmental stage E14.5. To contrast the blood vasculature, *Griffonia simplicifolia* isolectin (IB4), coupled to Alexa Fluor 647 fluorescent dye, was infused into the vitelline vein of the explanted conceptus. After placenta and yolk sac were dissected away, the fetus was formaldehyde fixed and cleared following the CUBIC protocol. LSFM provided a 3D reconstruction of the tdTomato-labeled lymphatic vasculature in the head of the fetus, shown in Figure 5A and Video S1 as a volume rendering, of the individual and merged vascular systems. While CUBIC clearing provided the optimal results with respect to fluorescent protein preservation, it remained inferior to organic solvent clearing with regarding optimal sample clarity and imaging quality. This is demonstrated by comparison of the IB4 Alexa Fluor 647-contrasted blood vasculature in a fetus of the same developmental stage cleared by methanol/BABB. An individual LSFM sample plane and a maximum intensity projection of the digital volume rendering and demonstrating superior image quality are shown in Figure 5B and Video S2, respectively.

Video S1. Visualization of developing lymphatic vessels in the head of a mVegfr3-tdTomato E14.5 mouse fetus, related to Figure 7A Shown is a 3D reconstruction of developing cranial blood (contrasted by IB4-Alexa Fluor™ 647, green) and lymphatic vessels (endogenous fluorescence, red) of an optically sectioned embryo at E14.5 after CUBIC-based tissue clearing.

Video S2. Light sheet microscopic visualization of the blood vasculature of an E14.5 mouse fetus, related to Figure 5B Shown is a 3D reconstruction of IB4-Alexa Fluor™ 647-contrasted blood vessels of an E14.5 mouse fetus after BABB-based tissue clearing.

Taken together, while hydrophilic tissue clearing is clearly superior to organic solvent clearing with regard to the retention of the native signal of fluorescent proteins, organic clearing methods provide better imaging quality as they result in higher optical transparency.

### Semi-automatic segmentation and analysis of vascular networks in large light sheet microscopy-based data sets

The analysis of large tissue volumes up to entire organisms by LSFM gains enormous power through the capacity to analyze the spatial distribution of multiple molecular markers by multi-color signal acquisition. This results inevitably in the generation of large data sets typically in the range of several hundred gigabytes up to terabytes. Visualization and analysis of such large LSFM data demands powerful software and hardware. We have developed a hierarchical random walker approach for the semi-automatic segmentation of vessels with varying calibers in LSFM data sets. An organ that exemplifies the underlying difficulty particularly well is the kidney, where vascular staining highlights the increasingly finely branched vascular tree from the renal artery and vein to the level of the glomeruli, where the fine vessels of the vascular tufts are often approaching or below the resolution limit of LSFM. Due to their lumen, vessels appear as double lines of various distances. Completely automatic segmentation of “hollow” vessel images is difficult; we, therefore applied an arbitrary, user-guided segmentation technique, an extension of the random walker method by Grady (Grady, 2006). In the basic method, each voxel of the input volume is assigned a foreground probability based on proximity to a set of user-provided foreground and background seeds and depending on image features in between. The probability of unlabeled voxels belonging to the foreground of the image is calculated, and a binary segmentation is obtained by thresholding the probability image.

While this standard method is popular in biomedical applications, it is not suitable for large volume or multiscale analysis due to high main or graphics memory and computational requirements. On a graphics card with 6 GB memory, we were only able to process volumes up to a size of roughly 100–400 MB with a computation time of several minutes using the conjugate gradient for solving the linear equation system. It is possible to obtain larger vessel structures through downsampling the image and separately analyzing a small sample of the capillary network by cropping to a region of interest. However, that approach does not achieve a full multiscale analysis.

To be able to analyze large volumes at multiple scales of resolution, we developed a hierarchical framework (Drees and Xiaoyi, 2021) that integrates the random walker method where in a preprocessing step, an octree LOD (level-of-detail) pyramid as commonly used for raycasting rendering of large data sets (Crassin et al., 2009) is created. In this approach, each layer is subdivided into cubic 32 × 32 × 32 voxel blocks, and blocks of a higher level in the pyramid are generated by combining half-sampling 2 × 2 × 2 higher resolution blocks, i.e., obtaining a new block voxel by averaging the intensity of 2 × 2 × 2 neighboring voxels. This is repeated until only a single, low-resolution root block remains. The standard random walker segmentation method as described above is then applied to blocks, first at lower resolution layers starting from the root block and then at higher resolution layers. The output foreground probabilities of a lower resolution act as (non-binary) seeds at the border of the current block. This way, the general shape of structures is propagated from lower resolution levels, and higher resolution levels are used to refine the border regions of the structure of interest. This was combined with pruning of octree branches, where the foreground/background class was already determined because blocks lay completely within the foreground structure or within the background. Additionally, octree branches from the previous segmentation were reused if the foreground probability map of a block had not changed significantly. This reduced the response times of the algorithm to the order of seconds in many cases, thus making interactive segmentation feasible.

**Figure 6:**
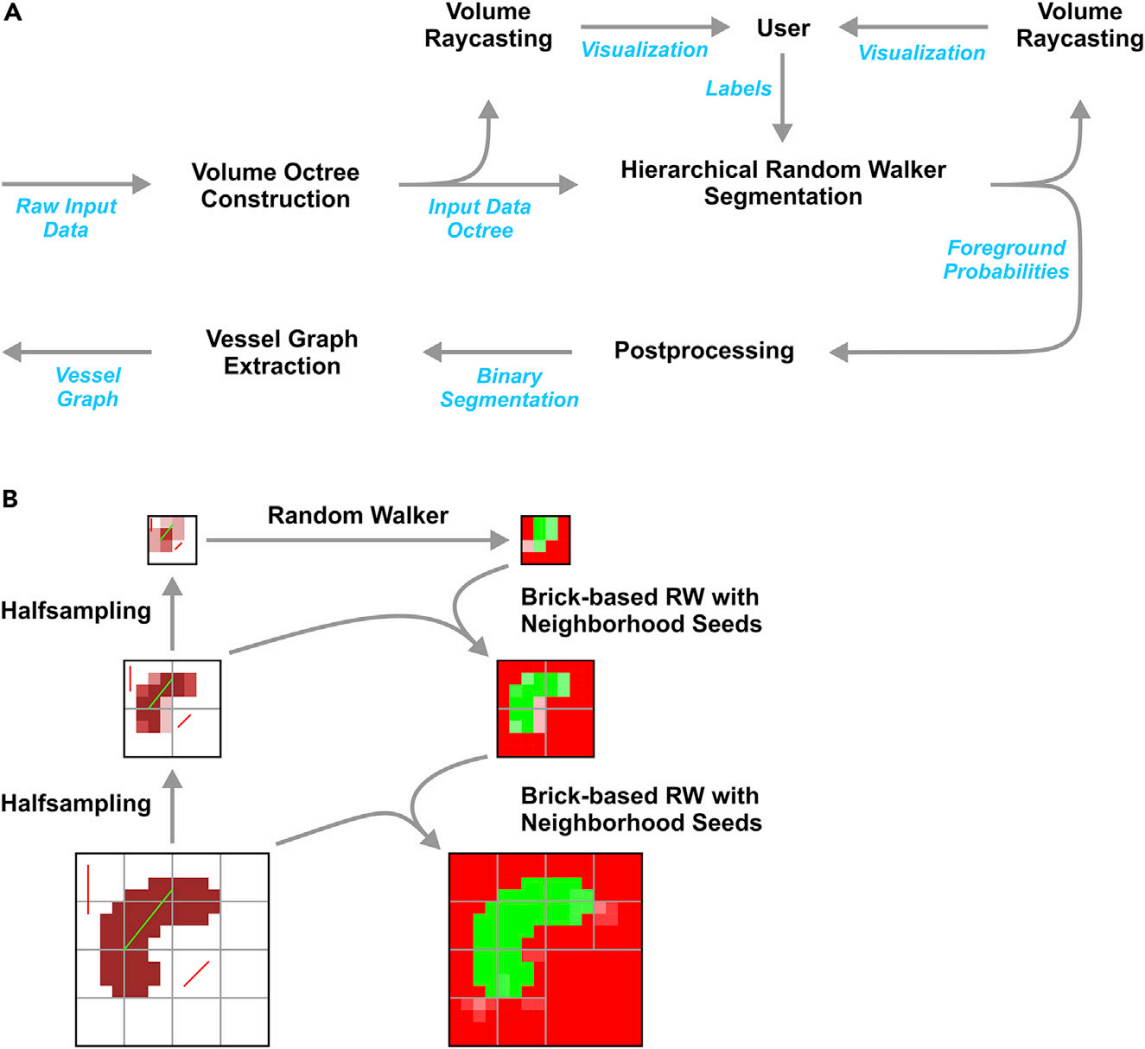
Workflow of a hierarchical random walker segmentation-based pipeline for the analysis of vascular structures in large multiscale image data sets (A) In a first step, a volume octree is generated from the input to visualize the image data virtually in 3D using the volume raycaster Voreen. The user then initiates the semi supervised hierarchical random walker segmentation by interactive, manual placement of seeds in the visualized volume. Under continuous visual inspection, the process is reiteratively repeated until a sufficient marking depth is reached. After determination of the foreground probability, the algorithm performs the necessary computational steps to complete the binary segmentation, from which a vessel graph extraction for visual output is performed and the segmentation result is displayed overlaid onto the original data. (B) 2D Illustration of the hierarchical segmentation method: First, the original volume (bottom left) is divided into bricks and then half-sampled to create the octree representation that is also used for rendering. User provided labels (red/green lines) are scaled so that they can be used at any resolution level. An initial foreground probability map is created on the coarsest level consisting of a single brick by applying the standard random walker method using the image and scaled labels. Then, the probability map is refined top-down, layer-by-layer, brick-after-brick, using the original image bricks, user provided labels, and continuous labels sampled from the next coarser probability map. For homogeneous bricks (bottom right), no further refinement is necessary.

Segmentations of vessel structures can then be processed further to derive a graph structure with centerlines of all vessel segments and nodes at branching points. The applied graph extraction method is applicable to out-of-core and multiscale data sets (Drees et al., 2021). The workflow of the proposed method is shown in Figure 6.

Application on multiscale data sets is enabled by “bulge size” as a per vessel segment property, which defines how far a vessel reaches out from a branching point of the parent vessel. It is computed by dividing its proper length (total length minus parent vessel radius at the bifurcation) by its diameter at the bifurcation. A threshold for this parameter is used during a pruning step to simplify the extracted vessel topology and remove spurious branches. In contrast to other commonly used properties used for pruning (such as absolute vessel length), it is dimensionless and independent of the true vessel size, thus making it suitable for multiscale data sets that contain vessels with radii spanning multiple order of magnitudes.

**Figure 7:**
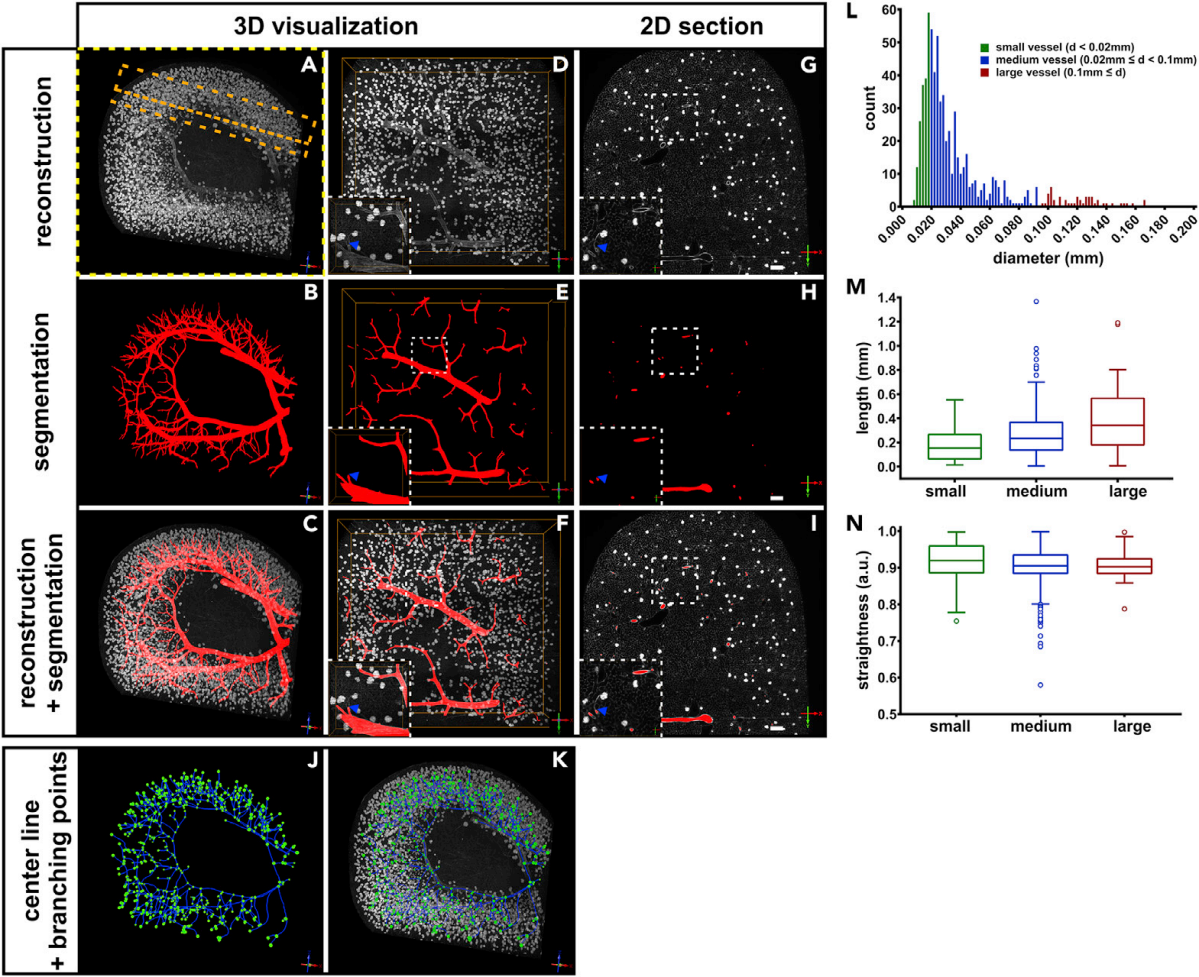
Hierarchical random walker segmentation of a subvolume of the renal blood vasculature in 3D volume reconstructions (A–C) (A) Coronal volume reconstruction of the blood vessels in a subvolume of a mouse kidney. The underlying data set comprised 60 GB and corresponded to physical dimensions of 4.0 × 3.6 × 3.6 mm (indicated by the yellow rectangle in Figure 8). (B) The hierarchical random walker segmentation of the renal blood vessels to the level of the interlobular vasculature. (C) Combined view of PECAM-1 immunostaining and vessel graph. (D–F) Axial view of a 750-μm slice obtained from the volume indicated by the orange rectangle in (A). Inset depicts segmentation of a vessel leading toward the afferent arteriole of a glomerulus (blue arrowhead). (G–I) Single sectional plane of 3-μm thickness, again indicated by an orange line in (A). Scale bar represents 200 μm. (J and K) Centerline view of segmented vessels (blue lines) with vessel branching points (green dots). (L) Frequency distribution (bin width 2 μm) of the vessel diameter (d). Based on the diameter, segments were classified into small (d < 0.02 mm), medium (0.02 mm ≤ d < 0.1 mm), and large (0.1 mm ≤ d) sized vessels. (M and N) Vessel length (in mm) and straightness (arbitrary units) of the classified vessels segments extracted from the foreground segmentation. The line in the box-and-whisker plots represents the median, the boxes represent the upper and lower quartile, and the end of the whiskers represents the 1.5-fold interquartile range.

We applied this hierarchical random walker approach first to a 60 GB data set that covered 52 mm ^3^ of a BABB-cleared, anti-PECAM1-stained mouse kidney. Labeling of seeds was done in a similar fashion as described by Prassni et al. (Prassni et al., 2010) using a multi-view interface of the data: The input data set and the intermediate segmentation results were rendered in 3D using the OpenCL raycaster of Voreen (Figure 7 and Video S3) (Dierkes et al., 2018). Vessels of a total length of 160 mm enclosing a volume of 0.35 mm^3^ and comprising the renal vasculature to the level of the interlobular vasculature were segmented interactively (Figures 7B, 7E, and 7H). The total organ volume in the data set was 48 mm³. Interactive label time for the vessel tree was roughly 7 hr, while the organ volume segmentation required only approx. 20 minutes of user interaction.

Video S3. 3D volume reconstruction of a subvolume of the renal blood vasculature, related to Figure 7 Shown is a coronal volume reconstruction of the blood vessels in a subvolume of a mouse kidney with a combined view of PECAM-1 immunostaining (white) and vessel graph (red).

**Figure 8:**
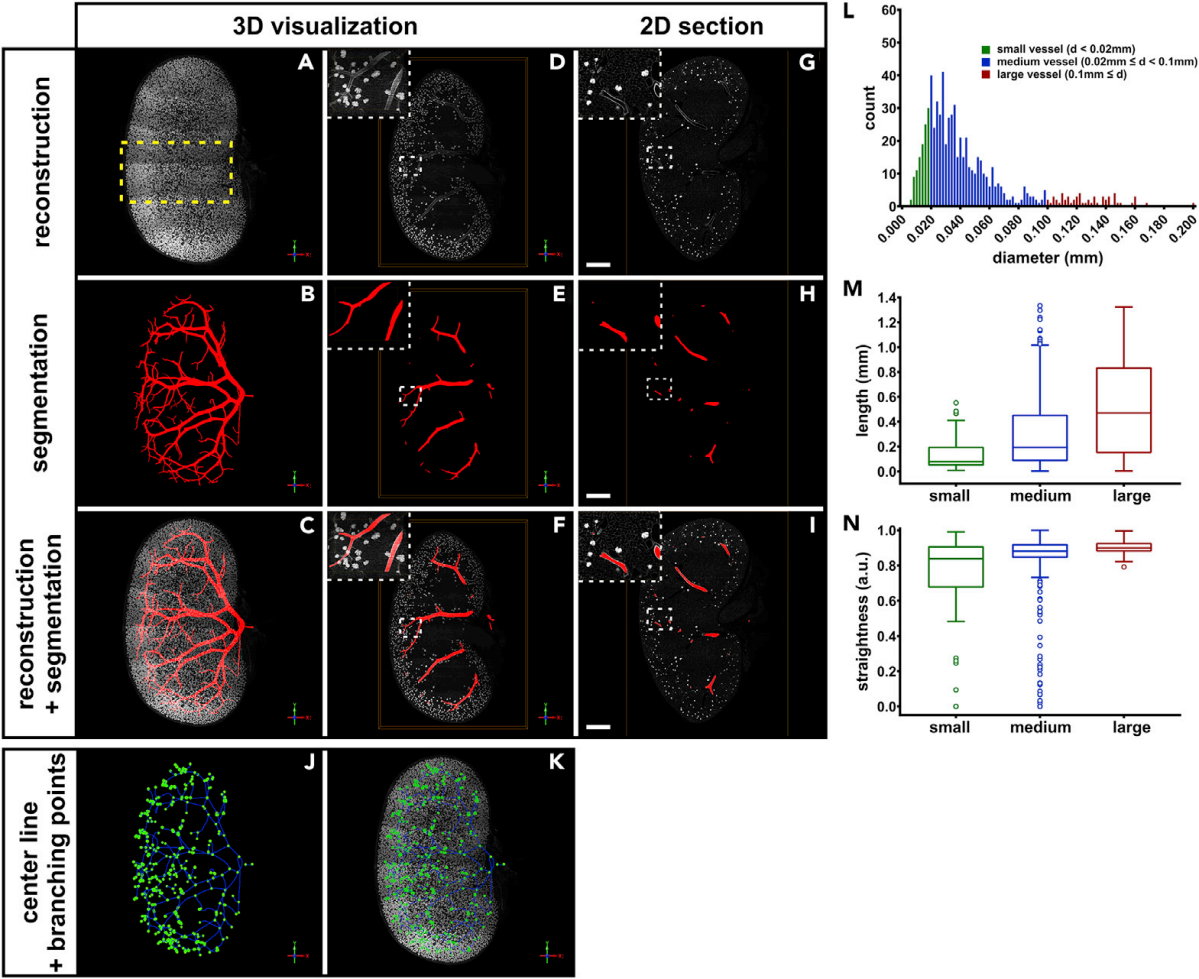
Hierarchical random walker segmentation of the renal blood vasculature in a 3D volume reconstruction of a complete mouse kidney (A–C) (A) Axial 3D reconstruction of the blood vasculature of large 6 x 5 multi-tile stitch volume image stack of 377 GB size. Shown is a complete mouse kidney (sample dimensions: 6.9 × 9.6 × 4.9 mm) wholemount immunostained for PECAM-1. The area corresponding to the subvolume analyzed in Figure 7 is marked by the yellow rectangle. (B) The resulting vessel graph of the hierarchical random walker segmentation depicting the renal blood vasculature to the level of the arcuate vessels. (C) Combined view of PECAM-1 immunostaining and vessel graph. (D–F) A 300 μm subvolume slice of the sample shown in (A–C) depicting the blood vessels below the renal cortex. Insets demonstrate the high level of detail of the data set. (G–I) Single sectional plane of 3-μm thickness. Scale bar represents 1 mm. (J and K) Centerline view of segmented vessels (blue lines) with vessel branching points (green dots). (L) Frequency distribution (bin width 2 μm) of the vessel diameter (d). Based on the diameter, segments were classified into small (d < 0.02 mm), medium (0.02 mm ≤ d < 0.1 mm), and large (0.1 mm ≤ d) sized vessels. (M and N) Vessel length (in mm) and straightness (arbitrary units) of the classified vessels segments extracted from the foreground segmentation. The line in the box-and-whisker plots represents the median, the boxes represent the upper and lower quartile, and the end of the whiskers represents the 1.5-fold interquartile range.

Having demonstrated the suitability of the approach for the interactive placement of labels in a large data set, we proceeded to a data set that represented an entire mouse kidney in a volume of 324 mm^3^ or 377 GB size, which greatly exceeded the available memory (16 GB). Figure 8 and Video S4 show the resulting segmentation, in which the renal vasculature to the level of the arcuate vessels was labeled. The segmented vasculature consisted of a total length of 164 mm, which enclosed a vessel volume of 0.67 mm^3^. In addition, the organ shape was also segmented using the presented approach, thus enabling the calculation of a total organ volume of 106 mm^3^. The user interaction labeling time of this data set was roughly 3 hr for the vascular tree and 30 min for the organ shape.

Video S4. 3D volume reconstruction of the renal blood vasculature of a complete mouse kidney, related to Figure 8 Shown is the 3D reconstruction of the blood vasculature of large volume image stack of the complete mouse kidney wholemount immunostained for PECAM-1 (white). The resulting vessel graph of the hierarchical random walker segmentation is shown in red.

Furthermore, the proposed segmentation method enables a quantitative evaluation of various parameters. Here, the diameter of each vessel segment was extracted and classified into small, medium, and large sized vessels (Figures 7 and 8L). We outlined two parameters: the vessel length as well as the vessel straightness of the classified vessel segments (Figurse 7, 8M, and 8N). As expected, the length of the vessel segments between branching points increases with its diameter, whereas the straightness is independent of the segment diameter.

## Discussion

Here, we present two additions to the light sheet microscopy toolbox: 3D-polymerized cell dispersions for the rapid testing of the capacity of fluorescent proteins to withstand tissue clearing and a semi-interactive method for image segmentation based on a hierarchical random walker algorithm.

Recently, LSFM-based volume imaging and analysis have been successfully used instead of histological sections in developmental and disease mechanism studies. Obvious advantages of LSFM over the analysis of serial sections include avoidance of the tissue loss and distortion associated with histological sectioning, superior spatial 3D digital reconstruction, and a high level of confidence in the detection of rare events. Due to the large data basis generated by volume imaging, infrequent or small parameter changes which can be difficult to identify in sections are detected with relative ease and high confidence making LSFM a promising technology for pathological analysis (Si et al., 2019; Pan et al., 2019; Hägerling et al., 2017). While dimensions up to one cm^3^ are already easily reached with existing light sheet microscopes, the desire to analyze larger tissue volumes for instance from human organs has sparked the development of a new microscope generation with the capacity to image even larger samples (Voigt et al., 2019, Zhao et al., 2020). The analysis of sample volumes up to several cm^3^ comprising entire organs or small organisms positions LSFM on the mesoscale, where it promises to bridge the gap between microscopic and whole body imaging technologies (Pan et al., 2016; Schoppe et al., 2020; Todorov et al., 2020; Kirst et al., 2020; Voigt et al., 2019).

The significant technical difficulties associated with whole-mount staining of entire organs or organisms are circumvented by genetically encoded reporter systems that tissue specifically express one or more of an increasing choice of fluorescent proteins (Macel et al., 2020). Applicability of fluorescent proteins may be limited by insufficient photostability or brightness, issues that have recently been addressed by the development of chemical-genetic hybrid systems that feature the combination of a genetically encoded high affinity tag with a highly tissue penetrable synthetic, organic fluorophore that may be systemically applied to ensure optimal distribution (Peresse and Gautier, 2019). The increasing variety of fluorescent labels is met by a multitude of tissue clearing techniques, which form the indispensable basis for large volume light sheet imaging. The choice of the optimal clearing protocol will in part be determined by the type of tissue under investigation and the degree of tissue clarity required. Soft tissues like the lung and brain are readily cleared by most protocols, while for heme-rich tissues like the liver or spleen, the decolorizing action of amino alcohols is preferable (Tainaka et al., 2016). Even hard tissues like long bones can be cleared satisfactorily using amino alcohols in combination with PEG stabilization (Jing et al., 2018).

An essential topic in the context of fluorescent proteins and tissue clearing that has so far often been neglected is the expression level of the reporter protein under scrutiny. Presently, most proof-of-principle studies have focused on transgenic model systems with robust expression like Thy-1-eGFP, Thy1-YFP, CAG-EGFP, or Ai14D mice (Feng et al., 2000; Liu et al., 2014; Madisen et al., 2010; Okabe et al., 1997), which have been widely used to demonstrate compatibility of fluorescent proteins with a particular tissue clearing protocol (Ertürk et al., 2012; Jing et al., 2018). It appears not farfetched that expression strength is an important parameter in the context of tissue clearing, as even a significant loss of protein fluorescence may appear acceptable if a reporter is massively expressed, while in case of moderate or low expression, the same protocol would be insufficient. Given the cost and complexity of the generation and maintenance of transgenic reporter animals, only few studies have comparatively addressed clearing protocols or transgenic models (Decroix et al., 2015, Kolesova et al., 2016, Orlich and Kiefer, 2018). This results in a pressing need for a rapid system to test the compatibility of fluorescent components and clearing chemistry, which is particularly obvious in view of the generation of transgenic model systems, where ill-informed choices lead to massive costs and loss of time.

To address this shortcoming, we introduced here 3D-polymerized cell dispersions, which are based on routine transfection procedures and low melting agarose, both inexpensive and readily available in many biomedical laboratories. Through variation of the embedded cell concentration or the use of transiently versus stably transfected cell lines, a wide range of expression strength can be simulated and evaluated. The approach is not limited to a single protein; multiple fluorescent proteins can be simultaneously analyzed. With little modification, 3D-polymerized cell dispersions can be applied to test the suitability of genetically encoded proteinaceous tags, which bind fluorophores or fluorogenic components or combined with antibody whole-mount staining protocols. We tested alternative polymer matrices including acrylamide or polydimethylsiloxane (data not shown); however, agarose was found to have the broadest applicability, as alternative polymers lost optical transmission in particular upon dehydration.

Preceding studies have only addressed the stability of one or two selected fluorescent proteins, and only a limited repertoire of clearing protocols was considered. Therefore, as shown here, 3D-polymerized cell dispersions allow the rapid comparison of any combination of fluorescent proteins and clearing protocols with a reasonable amount of time and money. In keeping with the available literature, organic-solvent-based tissue clearing methods like BABB and ECi were only recommendable for chemical fluorescent dye-stained samples where they excelled due to their superior and rapid clearing performance, while protein-based fluorescence was extinguished before clearing was completed. We selected BABB and ECi because both protocols are representative for other organic clearing protocols that use solvents with identical or similar chemical properties, like the 3DISCO (Ertürk et al., 2012) and iDISCO (Renier et al., 2014) protocols. The latter is tailored toward clearing of immunostained samples and does therefore not aim to retain the fluorescence of proteins. The single organic-based protocol that showed improved fluorescence retention in 3D-polymerized cell dispersions, however, which performed somewhat poorer in tissue samples was PEGASOS. Our analysis of cell populations expressing EGFP and tdTomato at different levels demonstrated that this was not due to selective loss of signal form weakly expressing samples. Relative signal preservation was comparable between weakly and strongly expressing cells; the counterintuitive seemingly enhanced fluorescence retention in low expressing cells was likely due to improved fluorescence detection with progressive tissue clearing. The less effective performance in tissue samples compared to 3D-polymerized cell dispersions may be due to interaction of tissue components like extracellular matrix proteins or myofibrils with the stabilizing factors like PEG or high pH. If retention of protein fluorescence was the primary goal, our analysis would suggest CUBIC as the protocol of choice for yielding a superior signal to noise ratio, albeit at the price of slightly reduced transparency and significantly longer incubation times. Finally, we have not considered protocols based on tissue transformation for instance through chemical co-polymer formation in this study because we found them to interfere significantly with antibody staining, which we consider as an indispensable prerequisite.

Multiscale biomedical image data sets are increasingly understood not simply as a basis for visual representation of the imaged volumes but as spatial databases, in which each voxel contains a set of parameters describing the cell or cells occupying this voxel. Beyond providing information about the containing voxel, these parameters are particularly valuable in the context of the properties of neighboring voxels, resulting in the need for approaches to segment and quantitate structures in light sheet microscopy-derived image stacks. To this end, fast parallel processing and machine learning approaches have been successfully employed to address this question, however, requiring substantial computing power (Kirst et al., 2020, Pan et al., 2019, Schoppe et al., 2020; Todorov et al., 2020) and reliable ground truth information for training. Yet there remains a need for approaches that are less demanding and allow the use of widely available consumer hardware. Here, we have taken an LOD pyramid-based approach for interactive segmentation of a vascular tree and have demonstrated its applicability to a PECAM1-stained, BABB-cleared mouse kidney data set of 377 GB. Despite the data set exceeding the main or graphics memory capacity by 24-fold, response times of the segmentation algorithm remained in the order of few seconds making interactive segmentation feasible. While the semi-automatic segmentation approach has the obvious downside of required user interaction resulting in a considerable workload, in particular if smaller vessels would have been segmented to completeness, the closed evaluation-edit-processing loop simultaneously integrates verification of the generated results. Furthermore, advanced human-machine interfaces like virtual reality packages or eye tracking could significantly reduce the user interaction time required. In effect, the generated segmentations are of very high quality such that the obtained results themselves can be understood as ground truth, which will be most valuable for future use as highly accurate training and validation data in fully automatic machine-learning-based segmentation approaches. In this sense, the presented method can be understood as an efficient labeling tool. Based on this, it may be worthwhile to explore how partial, semiautomatic segmentations (e.g., fully labeling a small region of the volume or more structured approaches such as fully labeling large diameter vessels and only labeling a small fraction of smaller vessels) can be extrapolated to full volume segmentations in order to reduce the required user interaction. Additionally, the method is also applicable and useful for post-processing of results obtained via fully automatic but less accurate segmentation methods or as an (interactive) segmentation method for use cases, which require a very reliable foreground segmentation, such as, for example, connectivity analysis enabled by the subsequent extraction of vessel topology, as demonstrated here. We have exemplified the suitability of the obtained data sets for analysis by vessel classification and extraction of the average vessel length and straightness, two immediately intuitive parameters, in the different vessel caliber classes. Other scenarios where such limited data sets may be valuable include studies that investigate and model flow and shear forces in vessels of a particular caliber or the relative position and distance of inflammatory foci in relation to particular vessel types or the volume of atherosclerotic lesions within the wall of arterial vessels of a particular caliber range.

The vessel graph construction method has a single parameter with an intuitive definition and can thus be considered unbiased. Due to its dimensionless nature, it can be applied for multiscale analysis, in contrast to approaches that employ vessel length for pruning. Especially for clearly defined, smooth surfaced vessels as in the presented example, the exact choice of value (3.0 here) is not critical.

Taken together, LSFM is in the process to become an indispensable tool in developmental biology, biomedical research, and pathology. The rapidly filling toolbox with 3D-polymerized cell dispersions and the here presented LOD pyramid-based interactive segmentation approach are new additions essentially contributing to this process.

### Limitations of the study

• The choice of polymer matrix is limited by the compatibility with clearing protocols, e.g., acrylamide or polydimethylsiloxan, which would better model more firm or harder tissues that are not compatible with dehydration by organic solvents.
• Autofluorescence of tissue components like the muscle of extracellular fibers is not modeled in 3D-polymerized cell dispersions.
• Due to the initial tissue opacity, fluorescence intensity can only be determined over a small volume during initial clearing stages, which may lead to an apparent increase in fluorescence with progressing clarity.
• The required user interaction time (interactive labeling) for segmentation of frequent fine structures in large data sets using the LOD pyramid-based interactive segmentation approach becomes prohibitively long.

## STAR★methods

### Key resources table

**Table:** 

REAGENT or RESOURCE	SOURCE	IDENTIFIER
**Antibodies**		

Rat monoclonal anti PECAM-1 (clone 5D2.6)	Wegmann et al., 2006	N/A
Rat monoclonal anti PECAM-1 (clone 1G5.1)	Wegmann et al., 2006	N/A

**Chemicals, peptides, and recombinant proteins**		

Isolectin GS-IB4 From Griffonia simplicifolia, Alexa Fluor™ 647 Conjugate	Invitrogen™	Cat# I32450; RRID: SCR_014365
UltraPure™ Low Melting Point Agarose	Invitrogen™	Cat# 16520100
Geneticin™ Selective Antibiotic (G418 Sulfate), Powder	Gibco™	Cat# 11811064
Benzyl alcohol	Sigma-Aldrich	Cat# 108006; CAS: 100-51-6
Benzyl benzoate, 99+%, ACROS Organics™	fisher scientific	Cat# 10607744
Ethyl cinnamate	Sigma-Aldrich	Cat# 112372; CAS: 103-36-6
tert-Butanol	Sigma-Aldrich	Cat# 308250; CAS: 75-65-0
Poly(ethylene glycol) methyl ether methacrylate	Sigma-Aldrich	Cat# 447943; CAS: 26915-72-0
N,N,N’,N’-Tetrakis(2-Hydroxypropyl)ethylenediamine (Quadrol)	Sigma-Aldrich	Cat# 122262; CAS: 102-60-3
Silicone oil	Sigma-Aldrich	Cat# 175633; CAS: 63148-52-7

**Experimental models: cell lines**		

Human: HEK 293T cells	ATTC	CRL-3216; RRID: CVCL_0063

**Experimental models: organisms/strains**		

Mouse: C57BL/6J	The Jackson Laboratory	JAX: 000664; RRID:IMSR_JAX:000664
Mouse: Lifeact-EGFP; Tg(CAG-EGFP)#Rows	Riedl et al., 2010	MGI:4831036
Mouse: m*Vegfr3*-tdTomato	Redder et al., 2021	N/A

**Recombinant DNA**		

pEGFP-C1	Clontech	N/A
pcDNA3.1-mOrange2	Roger Tsien (University of California, San Diego)	N/A
pcDNA3.1-mCherry	Kleaveland et al., 2018	RRID:Addgene_128744
tdTomato-N1	Shaner et al., 2004	RRID:Addgene_54642

**Software and algorithms**		

Fiji	Schindelin et al., 2012	https://fiji.sc/; *RRID:SCR_002285*
TeraStitcher	Bria and Iannello, 2012	https://abria.github.io/TeraStitcher/
Voreen	Meyer-Spradow et al., 2009	https://www.uni-muenster.de/Voreen/

### Resource availability

#### Lead contact

Further information and requests for resources and reagents should be directed to and will be fulfilled by the lead contact, Friedemann Kiefer (fkiefer@uni-muenster.de).

#### Materials availability

This study did not generate new unique wet-lab reagents.

#### Data and code availability

The data sets supporting the current study have not been deposited in a public repository but are available from the corresponding author on request.

### Experimental model and subject details

#### Mouse strains

Wild-type, Lifeact-EGFP^+/T^ (Riedl et al., 2010) and m*Vegfr3*-tdTomato^+/T^ (Redder et al., 2021) mouse embryos and adult mice of C57Bl/6 genetic background were analyzed at developmental stages 14.5 and at different ages (5 weeks – 1 year). Embryonic staging (E) was determined by the day of the vaginal plug (E0.5). All animal experiments were approved by the responsible animal committees at the sites of animal experimentation.

#### Cell lines

For stable cells lines were made HEK293T cells (RRID:CVCL_0063) were cultured at 37°C, 10% CO_2_ in DMEM supplemented with 10% FCS, 100 μg/ml penicillin and streptomycin, and 2 mM glutamine. Ca^2+^-phosphate co-precipitation (Sambrook and Russell, 2006) was used for transfections of mammalian expression plasmids encoding various fluorescent proteins (Table S1). Starting 48 hours after transfection, cells were selected with G418 sulfate

### Method details

#### Mouse embryo preparation

Pregnant dams were sacrificed by cervical dislocation and embryos collected in a petri dish containing cold phosphate buffered saline (PBS). Following two washes with ice cold PBS, embryos were fixed in 4% formaldehyde / PBS overnight at 4°C and washed twice with PBS and stored in PBS.

#### Murine tissue preparation

For tissue preparation, mice were sacrificed by cervical dislocation and diaphragm, kidney or lung tissue were dissected. Samples were washed twice with PBS, fixed in 4% formaldehyde / PBS for 4 hours at 4°C, again washed twice and stored in PBS.

#### Preparation of 3D-polymerized cell dispersions

HEK293T cells stably expressing fluorescent proteins were used for the preparation of 3D-polymerized cell dispersions. Cells were grown to confluency on 150 mm cell culture dishes. Then cells were detached from the culture dish with trypsin and pelleted by centrifugation for 10 minutes at 1300 rpm. About 4 x 10^7^ cells were resuspended in 4 mL prewarmed (37°C) 4% formaldehyde in PBS and fixed for 5 minutes at room temperature. The following steps were performed as quickly as possible to prevent premature curing of the agarose. After adding the same volume of prewarmed (37°C) 2% low-melting agarose (Invitrogen, Cat# 16520100), the suspension was mixed thoroughly. Finally, 1 mL of the cell agarose mixture was filled in a custom-made agarose mold. Molds of a size of 9 mm x 9 mm x 20 mm (see Figure S2) were 3D printed using synthetic resin. After hardening, the pellets were removed using a fitting 3D-printed stamp and stored until further use in PBS at 4°C. The 3D-polymerized cell dispersions had a final volume of 1.62 cm^3^ with a density of 3 x 10^3^ cells per mm^3^.

#### Immunofluorescence wholemount stainings

Prior to antibody staining, embryos and tissue were permeabilized (0.5% Triton X-100/PBS) and subsequently blocked in PermBlock solution (1% BSA, 0.5% Tween-20 in PBS) each for 1 day at 4°C. For wholemount stainings samples were incubated in rat monoclonal anti-mouse PECAM-1 [clone 5D2.6 and clone 1G5.1 (Wegmann et al., 2006)] antibody directly coupled to Alexa Fluor™ 647 dye or Isolectin GS-IB4 Alexa Fluor™ 647 conjugate (Invitrogen, Cat# I32450) diluted in PermBlock solution for 3 days at 4°C and subsequently were washed three times in PBS-T (0.5% Tween-20/PBS) each for 2 hours. Prior to tissue clearing, wholemount stained samples were embedded in 1% low-melting agarose to facilitate sample handling.

#### Tissue clearing

**BABB clearing** (Becker et al., 2008)

Samples were dehydrated in increasing concentrations of 50%, 70%, >99.5% and >99.5% (v/v) of methanol for at least 30 min each. After dehydration, tissues were incubated in a 1:1 mixture of >99.5% methanol and BABB (1:2 benzyl alcohol: benzyl benzoate) for 1 h and finally in BABB.

**ECi clearing** (Klingberg et al., 2017)

Samples were dehydrated in increasing concentrations of 30%, 50%, 70% (v/v) of ethanol adjusted to pH 9 and finally two times in >99.5% (v/v) ethanol for at least 30 min each. After dehydration, the samples were transferred to ECi (Sigma-Aldrich, Cat# 112372).

**PEGASOS clearing** (Jing et al., 2018)

For delipidation and dehydration, samples were placed in gradient tert-butanol solutions (30%, 50% and 70% tert-butanol (v/v), supplemented with 3% Quadrol (w/v)) for at least 30 min each and then in tert-butanol-polyethylene glycol (tB-PEG) solution (70% tert-butanol (v/v) + 30% PEG-MMA-500 (v/v) + 3% Quadrol (w/v)) twice for 1 h. For final clearing, samples were incubated into benzyl benzoate-polyethylene glycol (BB-PEG) clearing solution (75% benzyl benzoate (v/v) + 25% PEG-MMA-500 (v/v) + 3% Quadrol (w/v)) until transparency is reached.

**CUBIC clearing** (Susaki et al., 2014)

For composition of clearing solutions (reagent-1A and reagent-2) see Susaki et al. (2014) and Susaki and Ueda (2016) or http://cubic.riken.jp.

For clearing, samples were incubated in reagent-1A up to 4 days. while reagent-1A was replaced every 24 hours. Afterward samples were washed several times in PBS and were then incubated reagent-2 until final transparency. Before Imaging the samples were immersed in silicon oil (Sigma, Cat# 175633) for at least 1 h.

#### Widefield fluorescence microscopy

Imaging of murine tissue samples pre and post tissue clearing procedures was conducted on a Nikon Eclipse Ti2 inverted fluorescence microscope (Nikon, Japan) using a 10X Plan Fluor (NA 0.3, WD 15.20 mm) objective and appropriate emission filter sets for Cy3 (577/25 nm) and Cy5 (645/75 nm). The system is equipped with a SPECTRA X light engine® (Lumencor, USA) for excitation. The samples were placed into an 8 well chamber slide (Ibidi GmbH, Germany) and covered with the respective clearing medium before imaging.

#### Confocal laser scanning microscopy

High-resolution images of murine tissue samples were obtained with laser scanning microscope (LSM) 880 system (Zeiss, Germany) using a 10X Plan Apo (NA 0.45, WD 2.0 mm) objective. The samples were placed into an 8 well chamber slide (Ibidi GmbH, Germany) and covered with the respective clearing medium before imaging.

#### Light sheet fluorescence microscopy

Optically cleared samples were imaged on a LaVision BioTec Ultramicroscope II (LaVision BioTec, Bielefeld, Germany) with an Olympus MVX10 Zoom Microscope Body (Olympus, Tokyo, Japan) with an optical magnification range from 1.26x to 12.6x and an NA of 0.5. An NKT SuperK (Power SK PP485) supercontinuum white light laser served as excitation light source. For excitation and emission detection of specific fluorophores custom band-pass filters (excitation 470/40, 577/25 or 640/30 nm; emission 525/50, 632/60 or 690/50 nm) in combination with an Andor Neo sCMOS Camera with a pixel size of 6.5 x 6.5 μm^2^ were used. For image acquisition, Z-steps 3 μm were chosen and tile scanning was performed with an overlap of 20%.

### Quantification and statistical analysis

#### Fluorescence intensity measurements

3D-polymerized cell dispersions from different cell lines stably expressing various fluorescent proteins were used. Fluorescent images were captured using an Ultramicroscope II with the same imaging parameters for all testing groups. Z-step size was set to 10 μm and stack with a depth of 500 μm at magnification of 1.6X was acquired.

Afterward, fluorescence intensity values were measured using Fiji (Schindelin et al., 2012). For analysis of fluorescence preservation with different clearing methods, the initial time point (t0), was set immersing samples into PBS. Afterward, measurements for the “mean gray value” were made at indicated time points. The t0 fluorescence intensity value was normalized as 1.00 and the relative fluorescence intensity was shown as the ratio of fluorescence intensity over time to fluorescence intensity at t0.

#### Measurement of light transmission

Light transmission of 3D-polymerized cell dispersions during various tissue clearing methods was acquired using commercially available spectrophotometer (UV-1800, Shimadzu, Japan). 3D-polymerized cell dispersions were mounted in 10 mm high precision cells (Hellma Analytics, Deutschland) in the corresponding clearing solution and light transmission was measured at 561 nm. The final measuring value was normalized as 1.00 and the relative light transmission was shown as the ratio of light transmission over time.

#### Image analysis

Tile scanned light sheet imaging data were stitched using TeraStitcher (Bria and Iannello, 2012). Image processing and 3D reconstruction of (stitched) image stacks was performed using the volume visualization framework Voreen (Meyer-Spradow et al., 2009), which has been extended to allow the visualization, processing and analysis of large image stacks.

#### Statistical analysis

The mean of at least n=3 different images was calculated and statistically evaluated. Data are presented as mean ± SEM Statistical analysis was performed in Microsoft Excel and GraphPad Prism.

#### Image segmentation and analysis

For ease of use, the presented processing pipeline was integrated into the volume visualization and processing framework Voreen. The pipeline comprises 3 main steps: The semi-automatic segmentation of vessels, subsequent postprocessing as well as construction and analysis of a symbolic description of the segmented vessel tree. The segmentation is of general nature and was also be used to segment the organ volume.

The segmentation was done interactively using a hierarchical framework that allows application of the random walker image segmentation method to out-of-core data sets (Drees and Xiaoyi, 2021)(Grady, 2006). Intermediate segmentation results were visualized using uncertainty visualization and methods for the visualization of large image stacks (Prassni et al., 2010; Crassin et al., 2009).

The generated segmentation was postprocessed by smoothing the vessel surface with a median filter and removing cavities and spurious foreground fragments using a connected component analysis algorithm suitable for application on out-of-core data sets (Isenburg, 2011).

A graph representation with centerlines of vessels connecting nodes at the center of branching points is extracted from the high quality foreground segmentation of the vessel tree using an out-of-core procedure (Drees et al., 2021). An initial voxel skeleton is created using voxel thinning based on the definitions of Lee et al. and then pruned on the *scale-independent* property of bulge size until only a high quality graph representation of the vessel network remains (Lee et al., 1994). From this, a number of global properties such as total vessel length and volume are extracted.
