# Supplementary material for: Rapid methods for the evaluation of fluorescent reporters in tissue clearing and the segmentation of large vascular structures
Source: iScience. 2021 May 26;24(6):102650. doi: 10.1016/j.isci.2021.102650 (PMC8192726; doi:10.1016/j.isci.2021.102650)
Supplement: Document S1. Figures S1–S3 and Table S1 [file mmc1.pdf]

iScience, Volume *24*

Supplemental information

Rapid methods for the evaluation of fluorescent reporters in tissue clearing and the segmentation of large vascular structures

Nils Kirschnick, Dominik Drees, Esther Redder, Raghu Erapaneedi, Abel Pereira da Graca, Michael Schäfers, Xiaoyi Jiang, and Friedemann Kiefer

Table S1. Plasmids used in this study. Related to Figure 2.

Plasmid	Source
**pEGFP-C1**	Clontech Laboratories
**pcDNA3.1-mOrange2**	Kind gift of Roger Tsien (University of California, San Diego)
**pcDNA3.1-mCherry**	Addgene #128744
**tdTomato-N1**	Addgene #54642

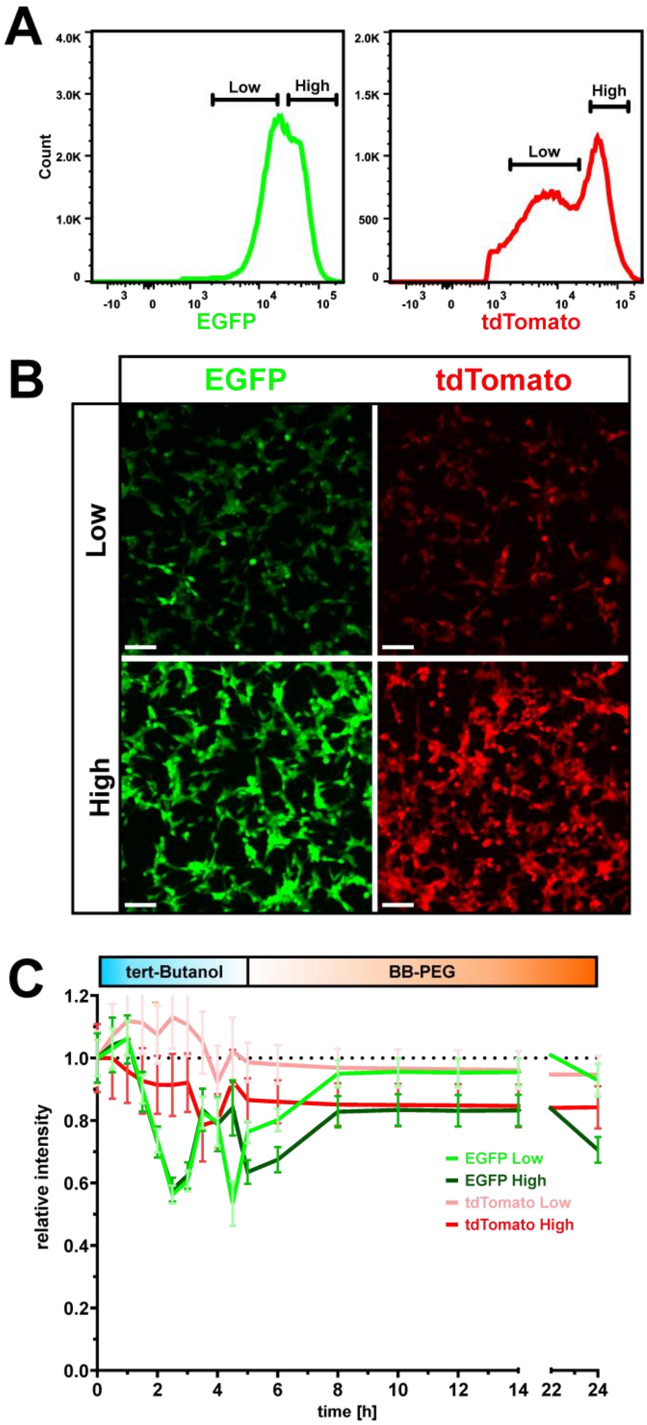

Figure S1: Effect of fluorescent protein expression level on fluorescence retention during PEGASOS clearing. Related to Figure 2. A) HEK293T cells were sorted by flow cytometry based on fluorescence intensity of either EGFP or tdTomato into low and high expressing populations. **B)** Representative epifluorescence images of sorted HEK293T cells. Scale bar = 100 µm. **C)** HEK293T cells stably expressing low or high levels of either EGFP or tdTomato were embedded in agarose and subjected to PEGAOS tissue clearing. Dehydration / delipidation in tert-butanol / Quadrol (blue box on top) followed by refractory index matching in benzyl benzoate / PEG-MMA-500 (BB-PEG) (orange box on top). Fluorescence intensities at t=0 were normalized to 1 and colored line plots denote the relative fluorescence intensity of the indicated proteins and levels in four independent samples (mean +/- SEM).

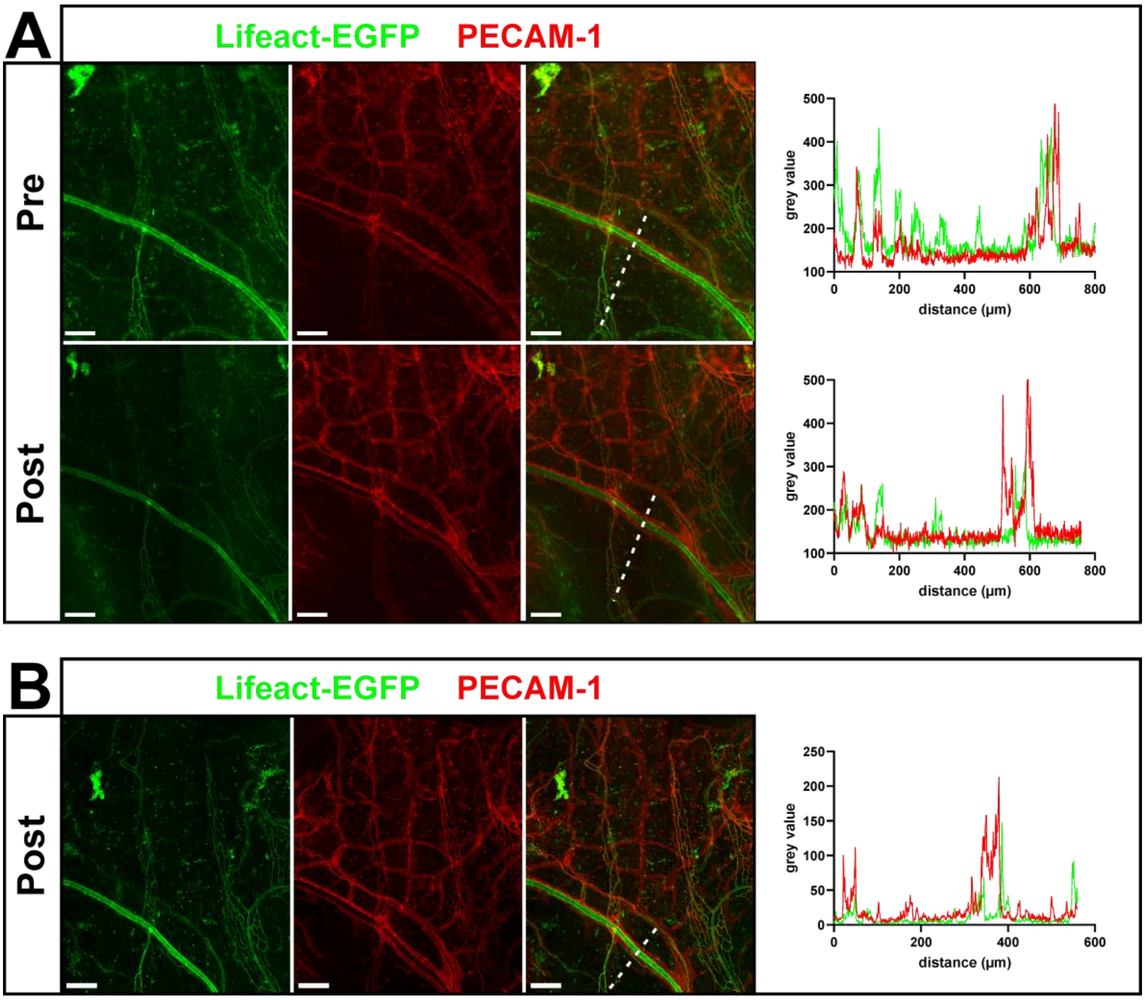

Figure S2: Fluorescence retention after PEGASOS clearing of the diaphragm of a Lifeact-EGFP reporter mouse. Related to Figure 3. A) The fixed diaphragm of a Lifeact-EGFP reporter mouse was cleared following the PEGASOS tissue clearing protocol. Top and bottom rows show comparable viewfields before (Pre) and after (Post) PEGASOS clearing acquired by epifluorescence microscopy using LED illumination. Contrasting of the vascular system by staining with Alexa Fluor™ 647-labelled anti PECAM1 antibodies served to provide unambiguous anatomical landmarks for orientation. Scale bars = 200 µm. **B)** After epifluorescence imaging the cleared, mounted samples were reanalyzed using CLSM and merged panel corresponding to the viewfields in A is shown as a MIP. The intensity profile (grey value) of EGFP (green) and PECAM-1 (red) expression along the white dashed line is presented in the graph on the right. Scale bars = 150µm.

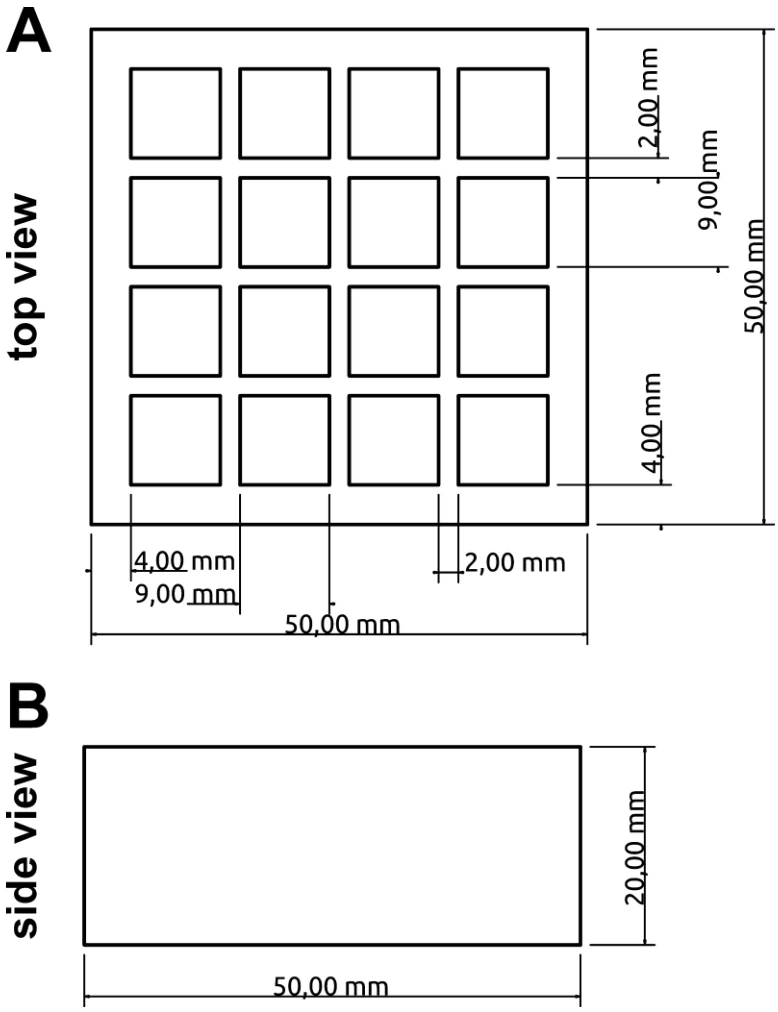

Figure S3: Technical drawing of the custom-made agarose mold displaying the top (A) and side (B) view with its physical dimensions. Related to Figure 1.
